# The Beneficial and Debilitating Effects of Environmental and Microbial Toxins, Drugs, Organic Solvents and Heavy Metals on the Onset and Progression of Multiple Sclerosis

**DOI:** 10.3390/toxins11030147

**Published:** 2019-03-05

**Authors:** Mahmood Y. Hachim, Noha M. Elemam, Azzam A. Maghazachi

**Affiliations:** Department of Clinical Sciences, College of Medicine, and the Immuno-Oncology group, Sharjah Institute for Medical Research (SIMR), University of Sharjah, Sharjah P.O. Box 27272, United Arab Emirates; noha.elemam211@gmail.com

**Keywords:** MS, toxins, EAE, environmental, natural, microbial

## Abstract

Multiple sclerosis (MS), a chronic inflammatory disease of the central nervous system is common amongst young adults, leading to major personal and socioeconomic burdens. However, it is still considered complex and challenging to understand and treat, in spite of the efforts made to explain its etiopathology. Despite the discovery of many genetic and environmental factors that might be related to its etiology, no clear answer was found about the causes of the illness and neither about the detailed mechanism of these environmental triggers that make individuals susceptible to MS. In this review, we will attempt to explore the major contributors to MS autoimmunity including genetic, epigenetic and ecological factors with a particular focus on toxins, chemicals or drugs that may trigger, modify or prevent MS disease.

## 1. Introduction

Multiple sclerosis (MS) affects the central nervous system ‘CNS’ that leads to focal plaques of primary demyelination and diffuse neurodegeneration in the grey and white matter of the brain and spinal cord [1]. Most MS cases are under the category of relapse-remitting disease that is, disease attacks are followed by recovery and stability periods [2]. Both pathological and radiological findings point to an early coexistence of neuroinflammation and neurodegeneration [3]. MS is one of the world’s most common neurologic disorders that leads to disability in young adults [4]. Approximately 2.5 million people worldwide suffer from MS, which is common in young adults, especially women [5]. This poses a major personal and socioeconomic burden with approximately 50% of patients requiring permanent use of a wheelchair [6]. MS is a complex and indecipherable disease and thought to be multifactorial, influenced by genetic as well as environmental factors [7]. Although an experimental model exists, it does not explain the variable clinical, pathological or immunological features of the disease [8]. MS management modalities have changed radically over the years to include not only disease-modifying therapies but also focusing on relapses and disability accrual prevention [9]. However, very little is known about the causes of MS disease. It remains much the same as during the time of William Boyd, who stated in 1958: “The amount of time and money which has been expended to determine the causal factors in multiple sclerosis is beyond computing … the result has been nil” [10].

## 2. Involvement of the Immune System in MS Disease

Immune mediated pathogenesis plays a significant role in MS [11], despite the strong barriers that restrict immune cells from reaching the immune-privileged CNS [12]. Whether MS is an autoimmune disease in its classical definition or inflammation associated demyelinating disease with some autoimmune manifestations remains a matter of controversy between different experts in the field, where each side showed their evidence in order to support or refute the autoimmune hypothesis [3]. 

The finding that half of MS immune function related genetic variants are shared with other putative autoimmune diseases makes the autoimmune model more acceptable [6,7]. Many recent publications referred MS as a prototypic autoimmune CNS disease [8,9,10], with autoimmune-mediated myelin injury in a susceptible host [11]. In such autoimmune model, autoreactive adaptive immune cells infiltrate and potentiate damage within the CNS [12]. CD4+ T cells are widely considered major players in the pathogenesis of MS [7]. CSF enrichment of functionally altered T helper cells (subtypes Th1 and Th17) and Treg cells as well as other leukocyte populations such as natural killer (NK) cells is documented in MS cases [13]. These lymphocyte skew towards a proinflammatory profile along with functional defects in the T and B regulatory subsets [14]. The autoreactive Th17 cells may pass the blood brain barrier or ‘BBB’ aided by IL-17 and IL-22 cytokines that disable tight junction proteins and endothelial cells, resulting in an influx of neutrophils leading to neuronal damage [15]. Pathogenic Th17 cells have low FasL expression, allowing them to escape the programmed cell death and persist in inflamed sites [16]. It was suggested that bystander activation upon viral infection can generate such autoreactive and potentially encephalitogenic T helper (Th)-1/17 cells which were recruited to the CSF following MS attacks [14]. Beside these identified players, new immune cells, like Interleukin (IL)-9 producing CD4+ T helper cells ‘Th9′ [17] and T helper 22 ‘Th22′ cells [18], which facilitate disease initiation or progress have been discovered. Moreover, recently CD8+ T cells were identified in MS lesions more than CD4+ T cells where their clones were found to be still present in blood and CSF after several years [7].

The study of B cell populations in MS plaques revealed an accumulation of clonally expanded B lymphocytes indicating the pivotal role of B cells, antibodies and its complement in the demyelination process [19]. Moreover, dendritic cells which act as antigen presenting cells (APCs) in addition to being effector cells in neuro-inflammation exacerbate MS pathology but the complete understanding of APCs role in human MS is still incomplete [7]. Based on these observations, the effect of environmental factors on genetically susceptible people is of immense need as it may initiate the cascade of damage. 

Despite the fact that MS is not an inherited disease, family case clustering of MS is prevalent amongst first-degree relatives who have similarities in their major histocompatibility complex (MHC) such as HLA DR15/DQ6 allele, alleles of interleukin-2 receptor alpha gene ‘IL2RA’ and interleukin-7 receptor alpha gene ‘IL7Rα’ [20]. Furthermore, some MS patients showed specific single-nucleotide polymorphisms in such genes [21]. It was also noted that these loci are related to the immune system and hence could possibly make individuals prone to autoimmune diseases. 

Genetic predisposition only explains a fraction of the disease risks [22]. Although by now more than a hundred genes are known to increase the risk of MS, these only contribute marginally [23]. Therefore, there must be another explanation for MS initiation. The parent-of-origin effect (i.e., the phenotypic effect of an allele depends on whether it is inherited from an individual’s mother or father) and the higher female-to-male ratio in MS are linked to the epigenetic X-chromosome inactivation and imprinting which have no susceptibility genes [21] indicating the importance of studying the epigenetic changes in such patients. In MS, epigenetic mechanisms are shown to affect T cell functions where histone acetylation was reported to occur in the white matter, hyper-methylation in oligodendrocyte survival genes and hypo-methylation in proteolytic processing genes [24]. Moreover, epidemiological data shows an interplay between genetic susceptibility and the environment by modulating the epigenome of the immune system [21]. Such epigenetic mechanisms readily respond to environmental factors [25,26]. 

**Figure 1:**
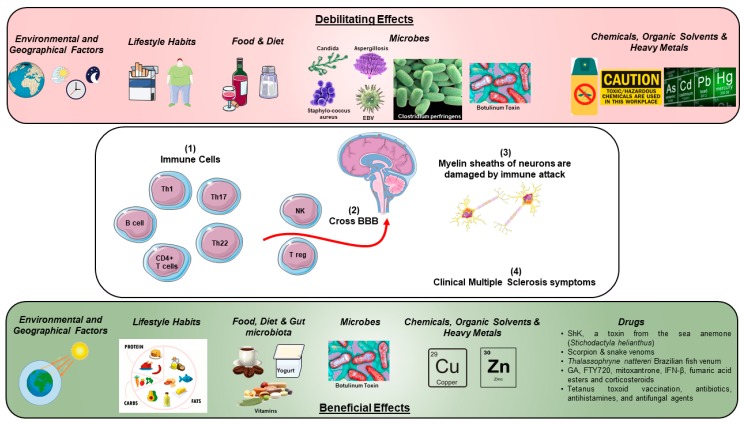
Beneficial and Debilitating Effects of Environmental and Microbial Toxins, Drugs, Organic Solvents and Heavy Metals on the Onset and Progression of Multiple Sclerosis.

Even though genetic or epigenetic factors may lead to autoimmunity, there might be an initial trigger that could help researchers understand the pathogenesis of the disease. A summary of such triggers that can have beneficial and debilitating effects on the onset and progression of multiple sclerosis is listed in Table 1 and illustrated in Figure 1. 

## 3. Toxic Effects of Environmental and Geographical Factors 

Environmental influences modify disease risk and progression, possibly through epigenetic changes which could up- or down-regulate the immune response and influence neural development [23,27]. Exposure to organic solvents, work shift, alcohol, high coffee consumption [22,28], infections, sun exposure/vitamin D and smoking were linked to MS disease development [29], nevertheless, there is still insufficient evidence to establish a causal role [30]. 

MS is unevenly distributed throughout the world and increases progressively with geographic latitude with pockets of high MS frequency [31]. People in certain communities showed concerns about clusters of MS; and the role of environmental elements in the development of the disease was investigated extensively, although no conclusion was reached [32]. For example, Key West in Florida has an unusually high prevalence of multiple sclerosis [33]. Also, MS is more prevalent in the northern parts of Great Britain and Northern Ireland than in England and Wales [34], suggesting strong links between geography and the incidence of this disease [35]. This is further supported by a study in Canada where MS prevalence differs according to the region, suggesting that these differences may be due to environmental factors [36]. On the other hand, some studies have reported that the north/south variation in the prevalence of MS could be possibly due to a change in the genetic predisposition of these populations to MS [37].

Among many environmental factors, sun exposure as a vitamin D source plays a vital role. There is a consistent finding in many epidemiological studies that the risk of MS is higher in areas with low levels of sun exposure and hence low vitamin D status [38,39], thus suggesting that vitamin D is a modifiable risk factor for MS [40]. This bolsters the idea of the protective effects of vitamin D intake on the risk of developing MS [41]. Studies reported that treatment with vitamin D_3_ improves clinical symptoms in the experimental autoimmune encephalomyelitis “EAE” mouse model [42]. It has been stated that low concentrations of neonatal vitamin D are associated with an increased risk of MS [43]. For instance, individuals born in November have significantly reduced incidence rate, linked to high levels of neonatal vitamin D exposure during the third trimester of pregnancy as a protective factor against multiple sclerosis [44]. Besides, vitamin D receptor (VDR) expression is hindered in MS and has been found to be regulated by the environment, genetics and epigenetics factors [45]. Increased vitamin D binding protein in the sera of MS patients exacerbate the pathophysiology of the disease [46]. It has been demonstrated that ultraviolet radiation may attenuate Th1-mediated immune responses [31] or may decrease the secretion of the immuno-stimulatory neurohormone melatonin from the pineal gland [47]. 

On the other hand, circadian disruption and sleep restriction can disturb the melatonin secretion and hence enhance pro-inflammatory responses. This might provide an explanation for multiple studies that link MS with age and work shifts [48,49], where a statistically significant association was reported between shift work at age 15–19 years and MS risk [50,51]. Hence, lifestyle and environmental factors are key contributors to the risk of MS [22]. Consequently, further research should focus on establishing the potential roots of MS disease by investigating the lifestyle habits (diet, physical activity) of patients and their role in the pathogenic pathways [29].

## 4. Toxic Effects of Lifestyle Habits 

An important risk factor for MS can be exposure to smoking [52] which may accelerate MS disease progression and disability [53]. Also, continued smoking is associated with an acceleration in time to secondary progressing MS [54]. The risk is further multiplied in HLA-DRB1*15 smokers due to a specific T-cell response to smoke that can aggravate the genetically regulated macrophage response [55]. Cigarette smoking is thus emerging as a modifiable risk factor for MS [56]. Family history of MS should be a warning sign for the family individual who smokes, where such a habit may aggravate or increase the chances of developing the disease [57].

On the other hand, there is strong evidence regarding the role of obesity during adolescence as a risk factor increasing MS [22,56]. There is also documented links between the incidence and severity of MS and fatty acids intake [58], as polyunsaturated fatty acids ‘PUFAs’ tend to reduce the frequency of relapses over two years [59]. Additionally, ketogenic diet can exert protective effects, likely via attenuation of the immune response and increased oxidative stress [60].

## 5. Toxic Effects of Food, Diet and Gut Microbiota

In studies of MS in animal model, high consumption of coffee was found to possibly decrease the risk of developing MS by suppressing the production of proinflammatory cytokines [61] due to the neuroprotective properties of caffeine [62]. Conversely, consumption of alcoholic beverages and fish are associated with the progression of the disability in relapsing onset MS disorder [63]. Likewise, high sodium intake can lead to an exaggerated clinical and radiological disease activity in patients with MS [64]. Besides the impact of vitamin D_3_ consumption, there is no strong evidence regarding the benefits or risks of other vitamins in the onset or progression of MS [65].

Furthermore, differences in diet, vitamin D_3_ insufficiency, smoking and alcohol consumption may affect the composition of the gut microbiota [66]. Gut microbiome is defined as all the microbial contents including genes, proteins and metabolic products in the gut at specific time [67]. Any disruption in the gut microbiota or so called “dysbiosis,” has been linked with several diseases [68]. In MS patients, the human gut microbiome exhibited variations in their composition and hence could be a cause or ameliorating agent in MS [69]. Furthermore, gut dysbiosis was found to increase intestinal and BBB permeability via microbiota-gut-brain axis, which could be restored upon intake of probiotics [70]. In this regard, improved hygiene influences autoimmune disorders highlighting the role and impact of gut flora on the development of EAE in mice [71]. 

## 6. Toxic Effects of Microbes

Water-damaged environments contain a complex mixture of contaminants produced by mould, Gram-negative and Gram-positive bacteria [72]. Such environment could be a site of chronic biotoxins that can lead to a cluster of MS-like illness cases [73]. Also, numerous infectious agents play a role in the onset of MS [74], as different viruses may trigger inflammatory demyelinating diseases resembling MS [75]. For instance, it has been reported that the timing of the primary Epstein Barr virus (EBV) infection at a certain age in individuals who are genetically susceptible, plays a major role in the development of the disease [76].

Bacterial toxins include staphylococcal, nasopharyngeal normal flora and many others that can distort immunity and cause toxic damage in the nervous system. It has been reported that staphylococcal toxins stimulate human T lymphocytes, leading to activation of the myelin autoantigens, the myelin basic protein and the proteolipid peptide. This results in reactive T lymphocytes that contribute to the demyelinating disease in humans [77]. It is worth mentioning that the CSF and extracellular fluid circulation are bi-directionally linked through a route by which products of nasopharyngeal infection may drain into the CNS and be processed by the immune cells of the meninges, which in turn may trigger brain damage [78]. Another bacterial toxin is the clostridium perfringens epsilon toxin ”ε-toxin,” where upon systemic administration, CNS white matter changes due to swelling of the myelin sheaths through the direct binding of ε-toxin to white matter. Blanch M et al. identified the myelin and lymphocyte (MAL) protein to be a key protein that could possibly mediate the cytotoxic effect of ε-toxin in inflammatory autoimmune diseases such as MS [79]. Furthermore, ε-toxin can cross the blood-brain barrier and precisely binds to myelinated fibres [80,81]. This leads to injury to oligodendrocytes or myelin, presenting a unique cellular target for ε-toxin in the CNS [82]. A study by Wagley et al. demonstrated an association between the presence of *Clostridium perfringens* ε-toxin and MS in the US population [83].

Pertussis toxin (PTX) and botulinum toxins are other bacterial toxins that have a great effect in MS. PTX exerts various effects in EAE mice, attenuating demyelination by about 75%. Furthermore, PTX reduces lymphocyte infiltration, deactivating microglia activation and changing T cell profile by increasing T helper type 1 and 2 as well as T regulatory cells [84]. Persistent PTX treatment is defensive from CNS autoimmune disease through upregulation of regulatory cytokines and expansion of CD4+CD25+FoxP3+ Treg cells. Multiple studies have reported that the bacteria-derived toxin, pertussis toxin, is known to lower susceptibility to EAE, despite the fact that its injection is needed to induce disease in some strains of mice [85,86]. On the contrary, botulinum toxin paralyzes muscles and is used as a traditional treatment of spasticity [87]. It was reported that this neurotoxin might improve the quality of life for many patients with MS [88,89].

Certain pathogenic fungi sequestered in non-neuronal tissues and release toxins that target astrocytes and oligodendrocytes causing myelin degradation and triggering MS [90]. Additionally, the secretion of different necrotizing factors in cerebral aspergillosis, can induce brain lesions and damage vital cells [91]. The food-associated mycotoxin ochratoxin A, exerts deleterious effects on numerous cell types including astrocytes [92]. Candida infection was found to be associated with increased odds of MS [93]. Several reports showed that MS patients might have antibodies against different Candida species [94], suggesting that this fungal infection may be a risk factor for MS [95]. Furthermore, C. Albicans infection prior to EAE induction in mice aggravates the disease, where a similar effect was found in MS patients [96]. Taking a closer look on the structural level, the insoluble N-acetyl-glucosamine polymer coating the fungal cell wall is usually hydrolysed by chitotriosidase ‘Chit,’ which is structurally homologous to chitinases [97]. These chitinases are synthesized and secreted by activated macrophages [98]. A study by Sotgiu S et al. revealed that the microglia-derived Chit activity in MS may protect the brain from the chitin-like substance deposition [97]. Moreover, increased Chit activity was demonstrated in the CNS of patients with different neurological disorders [98], as well as the plasma of MS patients [99]. Besides, it was reported that chitinases are increased in the CSF of patients with neuromyelitis optic in response to IL-13 thus leading to CNS inflammation through increased migration of immune cells across the blood-brain barrier [100]. Geographically, ergot fungi distribution showed a significant matching with the geographical distribution of MS [101]. Therefore, fungal infection might trigger multiple sclerosis or it may occur as a result of immune system dysfunction [102]. 

## 7. Toxicity of Chemicals, Organic Solvents and Heavy Metals

Exposure to chemicals, heavy metals and organic solvents are considered to be potential etiologic factors, contributing to the onset of MS in many studies [103], for example it was previously stated that tin, carbonic oxide and mercury but not zinc or manganese were considered to be the toxic causes of MS [104]. Areas with a high use of chemicals such as pesticides and mothballs have shown higher prevalence rates of MS [105,106,107,108]. Furthermore, workers engaged in agriculture who are exposed to pesticides, showed a higher risk of developing MS [109], particularly amongst women [110]. This could lead to an increased incidence in pregnant women and hence their babies could be more prone to MS [111]. Additionally, individuals who are subjected to chemicals such as workers in the shoe, leather and mechanical industries showed a higher risk of developing the disease [109,112]. Likewise, higher incidence of MS has been associated with areas highly polluted with heavy metals [113], such as Isfahan, the third largest city in Iran [114] and South-Western Sardinia, whereas the prevalence of MS is lower in areas of high mineral [115]. There is also a documented correlation between heavy metal imbalance and neurodegenerative pathologies [116], where toxic levels of metals when chelated from the serum led to an improvement of the MS disease status [117]. 

Mercury (Hg), a well-established toxicant, has been reported to be linked to autoimmunity [118] as it can induce oxidative stress as well as cause damage to DNA, mitochondria and lipid membranes [119]. Moreover, repeated exposure to mercury in animal subjects accelerated the progression of the disease through mitochondrial damage [120]. Just recently, a case of a man who injected himself with mercury provided a very good evidence that inorganic mercury is taken up by brain astrocytes, cortical oligodendrocytes, corticomotoneurons and locus coeruleus neurons. This might explain the involvement of mercury in MS and other CNS degenerative diseases [119]. Several reports have shown that the serum neuron-specific enolase (NSE-biomarker for the neurotoxic effects of mercury) is associated with the progression of multiple sclerosis [121]. Mercury-containing dental amalgam fillings increased the risk of MS [122] and was linked to neurobehavioral effects in dental personnel exposed to chronic low levels of mercury [123]. Despite these notes, data from patients with neurodegenerative diseases showed inconclusive data about a possible mercury involvement [124].

Another toxic heavy metal present in soils is lead that is associated with an increased incidence of MS, especially in males [125]. High lead toxicity and its ability to remain in the human body for prolonged time made it a suspect in the pathogenesis of many unexplained diseases [126]. The risk of MS was found to be increased 1.17 times per one µg/L increment of blood lead level [126]. However, another study stated that MS cases did not appear to cluster around lead smelters [32]. 

Arsenic is also present in soils and its exposure was also connected with MS disease, especially in females [125]. Consequently, arsenic may cause MS by inducing inflammation, degeneration and apoptosis of neuronal cells including hyperphosphorylation and aggregation of tau proteins leading to the deregulation of the tau function [127]. Quite the reverse, copper is used in the synthesis of myelin and hence its deficiency may potentially cause myelopathy [128]. Other metals show a controversial pattern literature such as zinc where lower serum levels were found in MS patients [129], while another study showed that zinc levels are increased in patients with MS. These results suggest that alterations of zinc concentrations may be involved in the pathogenesis of MS [130].

## 8. Efficacy of Various Drugs in MS Patients

For many years, several natural toxins have been described as therapeutic options for MS. Many novel compounds have been isolated from arthropods and other venomous animals for the treatment of major neurodegenerative diseases including MS [131]. These include ShK, a toxin from the sea anemone (*Stichodactyla helianthus*) and scorpion venom components, that are selective blockers of potassium channels needed for action of activated T lymphocytes [reviewed in 2]. Also, bee venom (from *Apis mellifera*) was found to ameliorate disease symptoms, improve motor function and reduce inflammatory markers [reviewed in 2]. Even snake venoms were found to play a vital role in MS therapy by inhibition of clinical signs of autoimmune encephalomyelitis and lymphocyte brain infiltration [132,133]. New molecules derived from the venom of *Thalassophryne nattereri* Brazilian fish, so called *TnP* family, generate systemic and CNS specific effects that result in inhibition of inflammatory leukocyte migration to CNS and demyelination and thus could be a therapeutic opportunity for the treatment of MS [134].

Several drugs and medications have been approved to reduce MS symptoms such as glatiramer acetate, fingolimod (FTY720), mitoxantrone, IFN-β, fumaric acid esters and corticosteroids [2,135,136]. Others have been proposed to reduce MS risks, such as tetanus toxoid vaccination, antibiotics, antihistamines and antifungal agents. However, their specific role remains to be validated [31]. Tetanus toxoid vaccination was reported to reduce the risk of MS by a third in vaccinated individuals compared with no vaccination [137]. Regarding antibiotics, a correlation between penicillin use and a lower risk of multiple sclerosis was described [138]. Other medications such as antihistamines may exhibit a possible beneficial effect, if introduced during the onset of MS [139], whereas the cholesterol-lowering statins were observed to prevent and reverse chronic and relapsing EAE [140]. The anti-seizure drug valproic acid (VPA) performs its action by increasing acetylated histone levels thus resulting in increased apoptosis in the neocortex and decreased cell proliferation in ganglionic eminence [141]. Moreover, VPA assists in remyelinating the lesions in MS through the introduction of endogenous progenitors [142] and may reduce spinal cord inflammation through apoptosis in activated T cells [143]. Additionally, VPA downregulates Th1 and Th17 cells and consequently, reduce the inflammatory cytokine levels [144].

Anti-inflammatory drugs were found to affect the immune system and thus could be of therapeutic value in MS. β-Amyrin, a cannabinoid receptor agonist, reduces inflammation in microglial cells and can be used as a potential anti-inflammatory agent in the CNS especially in neurodegenerative diseases. This drug affects the inflammatory mediators profile by reducing TNF-α, IL-1β, IL-6, PGE-2, COX-2 as well as the regulation of macrophage M1/M2 balance and the differentiation of microglia [145]. Another novel agent is WWL70, an anti-inflammatory therapeutic agent, that affects microglia in EAE mouse brain by reducing COX-2 and microsomal PGE_2_ expression [146]. A novel compound is JC-171 (a hydroxyl-sulphonamide analogue) that acts as a selective NLRP3 inflammasome inhibitor. In EAE mouse model, JC-171 was reported to hinder the progression and severity of the disease in both prophylactic and therapeutic experimental setups, thus encouraging its use in human MS [147]. Additionally, securinine, a major natural alkaloid product from the root of the plant *Securinega suffruticosa*, has been reported to have a potent biological activity via significant suppression of NO production in astrocytes and microglia as well as inhibition of the inflammatory mediator NF-κB and mitogen-activated protein kinases (MAPK). Therefore, it could be used as a potential therapeutic candidate for neuroinflammation related diseases [148].

Fumaric acid esters such as monomethyl fumarate (MMF) and dimethyl fumarate (DMF) have been intensively investigated over the last years. DMF has been approved for the treatment of various inflammatory mediated diseases including MS [149,150,151]. DMF has an immuno-modulatory function via shifting towards a Th2 cytokine profile and reducing the effect of Th1 and Th17 cells. More prominently, DMF and its metabolite MMF possess an antioxidant property by activating the nuclear factor (erythroid derived 2)-like2 (NRF2), thus stimulating cyto-protection of glial cells, oligodendrocytes and neurons [152,153]. DMF has been reported to affect the myeloid cells as well as lymphocytes including B cells and natural killer (NK) populations [154,155]. Our group investigated the effect of DMF and MMF on NK cells where we reported that they enhance the in vitro NK chemotaxis and cytolytic function [155,156,157]. MMF ameliorates EAE clinical score in mice by activating NK cells [42]. Fingolimod (Gilenya) also known as FTY720, a synthetic compound mimicking the fungal secondary metabolite myriocin (ISP-I), was reported to be a potent immuno-suppressant that was approved by the U.S. FDA as a therapeutic agent for MS [158]. It has been suggested that FTY720 affects the activity of immune cells such as NK cells via upregulating their activating receptors and potentiating their lytic activity against dendritic cells [159].

## 9. Conclusions

In this review, we shed some light on the diversity of chemicals, toxins and physical triggers in the environment that can affect the genetic and epigenetic composition of MS. These factors may modulate the function of immune players indirectly via modifying the body’s self-antigens that may be shared with CNS antigens or directly by unleashing the immune system to become unresponsive to inhibitory signals. The inconsistency of the epidemiological, experimental or clinical findings may be due to local and regional variations, both in the environment and population genetics.

## Figures and Tables

**Table 1:** Beneficial and Debilitating Effects of Environmental and Microbial Toxins, Drugs, Organic Solvents and Heavy Metals on the Onset and Progression of Multiple Sclerosis.

Environmental and Microbial Toxins, Drugs, Organic Solvents and Heavy Metals	Beneficial Effect	Debilitating Effect
**1. Environmental and Geographical Factors**		
• Geographic latitude		Yes
• Sun exposure as a vitamin D source	Yes	
• Circadian disruption and sleep restriction		Yes
**2. Toxic Effects of Lifestyle Habits**		
• Smoking		Yes
• Obesity and fatty acids intake		Yes
• Ketogenic diet	Yes	
**3. Toxic Effects of Food, Diet and Gut Microbiota**		
• Coffee	Yes	
• Alcoholic beverages and fish		yes
• High sodium intake		Yes
• Vitamins	Yes	
• Probiotics	Yes	
**4. Toxic Effects of Microbes**		
• Epstein Barr virus (EBV) infection		Yes
• Bacterial toxins include staphylococcal, nasopharyngeal normal flora		Yes
• Clostridium perfringens epsilon toxin” ε-toxin,”		Yes
• Pertussis toxin (PTX) and botulinum toxins	Yes	? Yes
• Aspergillosis		Yes
• Mycotoxin ochratoxin A		Yes
• Candida species		Yes
**5. Chemicals, Organic Solvents and Heavy Metals**		
• Pesticides and mothballs		Yes
• Occupational chemical exposure		Yes
• Heavy metals (mercury, lead, arsenic)		Yes
• Copper	Yes	
• Zinc	Yes	Yes
**6. Drugs**		
• ShK, a toxin from the sea anemone (*Stichodactyla helianthus*) and scorpion venom	Yes	
• Snake venoms	Yes	
• *Thalassophryne nattereri* Brazilian fish venom	Yes	
• Glatiramer acetate, fingolimod (FTY720), mitoxantrone, IFN-β, fumaric acid esters and corticosteroids	Yes	
• Tetanus toxoid vaccination, antibiotics, antihistamines and antifungal agents	Yes	

## References

[1] Mahad D.H., Trapp B.D., Lassmann H. (2015). Pathological mechanisms in progressive multiple sclerosis. Lancet Neurol..

[2] De Souza J.M., Goncalves B.D.C., Gomez M.V., Vieira L.B., Ribeiro F.M. (2018). Animal toxins as therapeutic tools to treat neurodegenerative diseases. Front. Pharmacol..

[3] Zeller D., Classen J. (2014). Plasticity of the motor system in multiple sclerosis. Neuroscience.

[4] Browne P., Chandraratna D., Angood C., Tremlett H., Baker C., Taylor B.V., Thompson A.J. (2014). Atlas of multiple sclerosis 2013: A growing global problem with widespread inequity. Neurology.

[5] Dilokthornsakul P., Valuck R.J., Nair K.V., Corboy J.R., Allen R.R., Campbell J.D. (2016). Multiple sclerosis prevalence in the united states commercially insured population. Neurology.

[6] Dendrou C.A., Fugger L., Friese M.A. (2015). Immunopathology of multiple sclerosis. Nat. Rev. Immunol..

[7] Adamczyk B., Adamczyk-Sowa M. (2016). New insights into the role of oxidative stress mechanisms in the pathophysiology and treatment of multiple sclerosis. Oxid. Med. Cell Longev..

[8] Lassmann H., Bradl M. (2017). Multiple sclerosis: Experimental models and reality. Acta Neuropathol..

[9] Comi G., Radaelli M., Soelberg Sørensen P. (2017). Evolving concepts in the treatment of relapsing multiple sclerosis. Lancet.

[10] Murray T.J. (2009). The history of multiple sclerosis: The changing frame of the disease over the centuries. J. Neurol. Sci..

[11] Gu C. (2016). Kir4.1: K(+) channel illusion or reality in the autoimmune pathogenesis of multiple sclerosis. Front. Mol. Neurosci..

[12] Høglund R.A., Maghazachi A.A. (2014). Multiple sclerosis and the role of immune cells. World J. Exp. Med..

[13] Maghazachi A.A. (2013). On the role of natural killer cells in neurodegenerative diseases. Toxins (Basel).

[14] Jones A.P., Kermode A.G., Lucas R.M., Carroll W.M., Nolan D., Hart P.H. (2016). Circulating immune cells in multiple sclerosis. Clin. Exp. Immunol..

[15] Jadidi-Niaragh F., Mirshafiey A. (2011). Th17 cell, the new player of neuroinflammatory process in multiple sclerosis. Scand. J. Immunol..

[16] Volpe E., Sambucci M., Battistini L., Borsellino G. (2016). Fas–fas ligand: Checkpoint of t cell functions in multiple sclerosis. Front. Immunol..

[17] Elyaman W., Khoury S.J. (2017). Th9 cells in the pathogenesis of eae and multiple sclerosis. Semin. Immunopathol..

[18] Fard N.A., Azizi G., Mirshafiey A. (2016). The potential role of t helper cell 22 and il-22 in immunopathogenesis of multiple sclerosis. Innov. Clin. Neurosci..

[19] Hestvik A.L.K. (2010). The double-edged sword of autoimmunity: Lessons from multiple sclerosis. Toxins (Basel).

[20] Garg N., Smith T.W. (2015). An update on immunopathogenesis, diagnosis, and treatment of multiple sclerosis. Brain Behav..

[21] Huynh J.L., Casaccia P. (2013). Epigenetic mechanisms in multiple sclerosis: Implications for pathogenesis and treatment. Lancet Neurol..

[22] Olsson T., Barcellos L.F., Alfredsson L. (2016). Interactions between genetic, lifestyle and environmental risk factors for multiple sclerosis. Nat. Rev. Neurol..

[23] Riemann-Lorenz K., Eilers M., von Geldern G., Schulz K.-H., Köpke S., Heesen C. (2016). Dietary interventions in multiple sclerosis: Development and pilot-testing of an evidence based patient education program. PLoS ONE.

[24] Peedicayil J. (2016). Epigenetic drugs for multiple sclerosis. Curr. Neuropharmacol..

[25] Babenko O., Kovalchuk I., Metz G.A. (2012). Epigenetic programming of neurodegenerative diseases by an adverse environment. Brain Res..

[26] Koch M.W., Metz L.M., Kovalchuk O. (2012). Epigenetic changes in patients with multiple sclerosis. Nat. Rev. Neurol..

[27] Miller F.W., Alfredsson L., Costenbader K.H., Kamen D.L., Nelson L.M., Norris J.M., De Roos A.J. (2012). Epidemiology of environmental exposures and human autoimmune diseases: Findings from a national institute of environmental health sciences expert panel workshop. J. Autoimmun..

[28] Hedström A.K., Alfredsson L., Olsson T. (2016). Environmental factors and their interactions with risk genotypes in ms susceptibility. Curr. Opin. Neurol..

[29] Kakalacheva K., Lünemann J.D. (2011). Environmental triggers of multiple sclerosis. FEBS Lett..

[30] Loken-Amsrud K.I., Lossius A., Torkildsen O., Holmoy T. (2015). Impact of the environment on multiple sclerosis. Tidsskr. Nor. Laegeforen..

[31] Milo R., Kahana E. (2010). Multiple sclerosis: Geoepidemiology, genetics and the environment. Autoimmun. Rev..

[32] Turabelidze G., Schootman M., Zhu B.P., Malone J.L., Horowitz S., Weidinger J., Williamson D., Simoes E. (2008). Multiple sclerosis prevalence and possible lead exposure. J. Neurol. Sci..

[33] Helmick C.G., Wrigley J.M., Zack M.M., Bigler W.J., Lehman J.L., Janssen R.S., Hartwig E.C., Witte J.J. (1989). Multiple sclerosis in key west, florida. Am. J. Epidemiol..

[34] Forbes R.B., Wilson S.V., Swingler R.J. (1999). The prevalence of multiple sclerosis in tayside, scotland: Do latitudinal gradients really exist?. J. Neurol..

[35] Gray O.M., McDonnell G.V., Hawkins S.A. (2008). Factors in the rising prevalence of multiple sclerosis in the north-east of ireland. Mult. Scler..

[36] Beck C.A., Metz L.M., Svenson L.W., Patten S.B. (2005). Regional variation of multiple sclerosis prevalence in canada. Mult. Scler..

[37] McGuigan C., McCarthy A., Quigley C., Bannan L., Hawkins S., Hutchinson M. (2004). Latitudinal variation in the prevalence of multiple sclerosis in ireland, an effect of genetic diversity. J. Neurol. Neurosurg. Psychiatry.

[38] Lucas R.M., Byrne S.N., Correale J., Ilschner S., Hart P.H. (2015). Ultraviolet radiation, vitamin d and multiple sclerosis. Neurodegener. Dis. Manag..

[39] Alharbi F.M. (2015). Update in vitamin d and multiple sclerosis. Neurosciences (Riyadh).

[40] Niino M., Sato S., Fukazawa T., Masaki K., Miyazaki Y., Matsuse D., Yamasaki R., Takahashi E., Kikuchi S., Kira J. (2015). Decreased serum vitamin d levels in japanese patients with multiple sclerosis. J. Neuroimmunol..

[41] Munger K.L., Zhang S.M., O’Reilly E., Hernan M.A., Olek M.J., Willett W.C., Ascherio A. (2004). Vitamin d intake and incidence of multiple sclerosis. Neurology.

[42] Al-Jaderi Z., Maghazachi A.A. (2015). Vitamin d3 and monomethyl fumarate enhance natural killer cell lysis of dendritic cells and ameliorate the clinical score in mice suffering from experimental autoimmune encephalomyelitis. Toxins (Basel).

[43] Nielsen N.M., Munger K.L., Koch-Henriksen N., Hougaard D.M., Magyari M., Jorgensen K.T., Lundqvist M., Simonsen J., Jess T., Cohen A. (2017). Neonatal vitamin d status and risk of multiple sclerosis: A population-based case-control study. Neurology.

[44] Fernandes de Abreu D.A., Babron M.C., Rebeix I., Fontenille C., Yaouanq J., Brassat D., Fontaine B., Clerget-Darpoux F., Jehan F., Feron F. (2009). Season of birth and not vitamin d receptor promoter polymorphisms is a risk factor for multiple sclerosis. Mult. Scler..

[45] Saccone D., Asani F., Bornman L. (2015). Regulation of the vitamin d receptor gene by environment, genetics and epigenetics. Gene.

[46] Rinaldi A.O., Sanseverino I., Purificato C., Cortese A., Mechelli R., Francisci S., Salvetti M., Millefiorini E., Gessani S., Gauzzi M.C. (2015). Increased circulating levels of vitamin d binding protein in ms patients. Toxins (Basel).

[47] Hutter C.D., Laing P. (1996). Multiple sclerosis: Sunlight, diet, immunology and aetiology. Med. Hypotheses.

[48] Hedstrom A.K., Akerstedt T., Hillert J., Olsson T., Alfredsson L. (2011). Shift work at young age is associated with increased risk for multiple sclerosis. Ann. Neurol..

[49] Ponsonby A.-L., Lucas R.M. (2011). Shift work and multiple sclerosis. Ann. Neurol..

[50] Gustavsen S., Sondergaard H.B., Oturai D.B., Laursen B., Laursen J.H., Magyari M., Ullum H., Larsen M.H., Sellebjerg F., Oturai A.B. (2016). Shift work at young age is associated with increased risk of multiple sclerosis in a danish population. Mult. Scler. Relat. Disord..

[51] Hedstrom A.K., Akerstedt T., Olsson T., Alfredsson L. (2015). Shift work influences multiple sclerosis risk. Mult. Scler..

[52] Zhang P., Wang R., Li Z., Wang Y., Gao C., Lv X., Song Y., Li B. (2016). The risk of smoking on multiple sclerosis: A meta-analysis based on 20,626 cases from case-control and cohort studies. PeerJ.

[53] Turner A.P., Hartoonian N., Maynard C., Leipertz S.L., Haselkorn J.K. (2015). Smoking and physical activity: Examining health behaviors and 15-year mortality among individuals with multiple sclerosis. Arch. Phys. Med. Rehabil..

[54] Ramanujam R., Hedström A., Manouchehrinia A., Alfredsson L., Olsson T., Bottai M., Hillert J. (2015). Effect of smoking cessation on multiple sclerosis prognosis. JAMA Neurol..

[55] Öckinger J., Hagemann-Jensen M., Kullberg S., Engvall B., Eklund A., Grunewald J., Piehl F., Olsson T., Wahlström J. (2016). T-cell activation and hla-regulated response to smoking in the deep airways of patients with multiple sclerosis. Clin. Immunol..

[56] Ascherio A., Munger K.L. (2007). Environmental risk factors for multiple sclerosis. Part ii: Noninfectious factors. Ann. Neurol..

[57] Hedström A.K., Bomfim I.L., Barcellos L.F., Briggs F., Schaefer C., Kockum I., Olsson T., Alfredsson L. (2014). Interaction between passive smoking and two hla genes with regard to multiple sclerosis risk. Int. J. Epidemiol..

[58] Timmermans S., Bogie J.F.J., Vanmierlo T., Lütjohann D., Stinissen P., Hellings N., Hendriks J.J.A. (2014). High fat diet exacerbates neuroinflammation in an animal model of multiple sclerosis by activation of the renin angiotensin system. J. Neuroimmune Pharmacol..

[59] Farinotti M., Vacchi L., Simi S., Di Pietrantonj C., Brait L., Filippini G. (2012). Dietary interventions for multiple sclerosis. Cochrane Database Syst. Rev..

[60] Kim D.Y., Hao J., Liu R., Turner G., Shi F.-D., Rho J.M. (2012). Inflammation-mediated memory dysfunction and effects of a ketogenic diet in a murine model of multiple sclerosis. PLoS ONE.

[61] Hedström A.K., Mowry E.M., Gianfrancesco M.A., Shao X., Schaefer C.A., Shen L., Olsson T., Barcellos L.F., Alfredsson L. (2016). High consumption of coffee is associated with decreased multiple sclerosis risk; results from two independent studies. J. Neurol. Neurosurg. Psychiatry.

[62] Mowry E., Hedstrom A., Gianfrancesco M., Shao X., Schaefer C., Barcellos L., Olsson T., Alfredsson L. (2015). Greater consumption of coffee is associated with reduced odds of multiple sclerosis (s45.004). Neurology.

[63] D’hooghe M.B., Haentjens P., Nagels G., De Keyser J. (2011). Alcohol, coffee, fish, smoking and disease progression in multiple sclerosis. Eur. J. Neurol..

[64] Farez M.F., Fiol M.P., Gaitán M.I., Quintana F.J., Correale J. (2015). Sodium intake is associated with increased disease activity in multiple sclerosis. J. Neurol. Neurosurg. Psychiatry.

[65] Riccio P., Rossano R. (2015). Nutrition facts in multiple sclerosis. ASN Neuro.

[66] Mielcarz D.W., Kasper L.H. (2015). The gut microbiome in multiple sclerosis. Curr. Treat. Opt. Neurol..

[67] Kirby T.O., Ochoa-Repáraz J. (2018). The gut microbiome in multiple sclerosis: A potential therapeutic avenue. Med. Sci. (Basel, Switzerland).

[68] Chu F., Shi M., Lang Y., Shen D., Jin T., Zhu J., Cui L. (2018). Gut microbiota in multiple sclerosis and experimental autoimmune encephalomyelitis: Current applications and future perspectives. Med. Inflamm..

[69] Jangi S., Gandhi R., Cox L.M., Li N., von Glehn F., Yan R., Patel B., Mazzola M.A., Liu S., Glanz B.L. (2016). Alterations of the human gut microbiome in multiple sclerosis. Nat. Commun..

[70] Roy Sarkar S., Banerjee S. (2019). Gut microbiota in neurodegenerative disorders. J. Neuroimmunol..

[71] Yokote H., Miyake S., Croxford J.L., Oki S., Mizusawa H., Yamamura T. (2008). Nkt cell-dependent amelioration of a mouse model of multiple sclerosis by altering gut flora. Am. J. Pathol..

[72] Brewer J.H., Thrasher J.D., Straus D.C., Madison R.A., Hooper D. (2013). Detection of mycotoxins in patients with chronic fatigue syndrome. Toxins (Basel).

[73] Ordog G. (2005). 476 multiple sclerosis cluster: Mycotoxic leukoencephalopathy. J. Investig. Med..

[74] Venkatesan A. (2015). Multiple sclerosis and infections. Neurodegener. Dis. Manag..

[75] Oleszak E.L., Chang J.R., Friedman H., Katsetos C.D., Platsoucas C.D. (2004). Theiler’s virus infection: A model for multiple sclerosis. Clin. Microbiol. Rev..

[76] Fong I.W. (2014). The Role of Microbes in Common Non-Infectious Diseases.

[77] Burns J., Littlefield K., Gill J., Trotter J.L. (1992). Bacterial toxin superantigens activate human t lymphocytes reactive with myelin autoantigens. Ann. Neurol..

[78] Gay F. (2007). Bacterial toxins and multiple sclerosis. J. Neurol. Sci..

[79] Blanch M., Dorca-Arévalo J., Not A., Cases M., Gómez de Aranda I., Martínez-Yélamos A., Martínez-Yélamos S., Solsona C., Blasi J. (2018). The cytotoxicity of epsilon toxin from clostridium perfringens on lymphocytes is mediated by mal protein expression. Mol. Cell. Biol..

[80] Cases M., Llobet A., Terni B., Gómez de Aranda I., Blanch M., Doohan B., Revill A., Brown A.M., Blasi J., Solsona C. (2017). Acute effect of pore-forming clostridium perfringens ε-toxin on compound action potentials of optic nerve of mouse. eNeuro.

[81] Uzal F.A., Navarro M.A., Li J., Freedman J.C., Shrestha A., McClane B.A. (2018). Comparative pathogenesis of enteric clostridial infections in humans and animals. Anaerobe.

[82] Linden J.R., Ma Y., Zhao B., Harris J.M., Rumah K.R., Schaeren-Wiemers N., Vartanian T. (2015). Clostridium perfringens epsilon toxin causes selective death of mature oligodendrocytes and central nervous system demyelination. mBio.

[83] Wagley S., Bokori-Brown M., Morcrette H., Malaspina A., D’Arcy C., Gnanapavan S., Lewis N., Popoff M.R., Raciborska D., Nicholas R. (2018). Evidence of clostridium perfringens epsilon toxin associated with multiple sclerosis. Mult. Scler. J..

[84] Yin J.-X., Tang Z., Gan Y., Li L., Shi F., Coons S., Shi J. (2014). Pertussis toxin modulates microglia and t cell profile to protect experimental autoimmune encephalomyelitis. Neuropharmacology.

[85] Weber M.S., Benkhoucha M., Lehmann-Horn K., Hertzenberg D., Sellner J., Santiago-Raber M.-L., Chofflon M., Hemmer B., Zamvil S.S., Lalive P.H. (2011). Repetitive pertussis toxin promotes development of regulatory t cells and prevents central nervous system autoimmune disease. PLoS ONE.

[86] Steelman A.J. (2015). Infection as an environmental trigger of multiple sclerosis disease exacerbation. Front. Immunol..

[87] Dressler D., Bhidayasiri R., Bohlega S., Chahidi A., Chung T.M., Ebke M., Jacinto L.J., Kaji R., Koçer S., Kanovsky P. (2017). Botulinum toxin therapy for treatment of spasticity in multiple sclerosis: Review and recommendations of the iab-interdisciplinary working group for movement disorders task force. J. Neurol..

[88] Cameron M.H., Bethoux F., Davis N., Frederick M. (2014). Botulinum toxin for symptomatic therapy in multiple sclerosis. Curr. Neurol. Neurosci. Rep..

[89] Latino P., Castelli L., Prosperini L., Marchetti M.R., Pozzilli C., Giovannelli M. (2017). Determinants of botulinum toxin discontinuation in multiple sclerosis: A retrospective study. Neurol. Sci..

[90] Purzycki C.B., Shain D.H. (2010). Fungal toxins and multiple sclerosis: A compelling connection. Brain Res. Bull..

[91] Speth C., Rambach G., Lass-Flörl C., Würzner R., Gasque P., Mohsenipour I., Dierich M.P. (2006). Culture supernatants of patient-derived aspergillus isolates have toxic and lytic activity towards neurons and glial cells. FEMS Immunol. Med. Microbiol..

[92] Razafimanjato H., Garmy N., Guo X.-J., Varini K., Di Scala C., Di Pasquale E., Taïeb N., Maresca M. (2010). The food-associated fungal neurotoxin ochratoxin a inhibits the absorption of glutamate by astrocytes through a decrease in cell surface expression of the excitatory amino-acid transporters glast and glt-1. Neurotoxicology.

[93] Benito-León J., Pisa D., Alonso R., Calleja P., Díaz-Sánchez M., Carrasco L. (2010). Association between multiple sclerosis and candida species: Evidence from a case-control study. Eur. J. Clin. Microbiol. Infect. Dis..

[94] Pisa D., Alonso R., Carrasco L. (2011). Fungal infection in a patient with multiple sclerosis. Eur. J. Clin. Microbiol. Infect. Dis..

[95] Pisa D., Alonso R., Jiménez-Jiménez F.J., Carrasco L. (2013). Fungal infection in cerebrospinal fluid from some patients with multiple sclerosis. Eur. J. Clin. Microbiol. Infect. Dis..

[96] Fraga-Silva T.F.C., Mimura L.A.N., Marchetti C.M., Chiuso-Minicucci F., França T.G.D., Zorzella-Pezavento S.F.G., Venturini J., Arruda M.S.P. (2015). Experimental autoimmune encephalomyelitis development is aggravated by candida albicans infection. J. Immunol. Res..

[97] Sotgiu S., Musumeci S., Marconi S., Gini B., Bonetti B. (2008). Different content of chitin-like polysaccharides in multiple sclerosis and alzheimer’s disease brains. J. Neuroimmunol..

[98] Barone R., Sotgiu S., Musumeci S. (2007). Plasma chitotriosidase in health and pathology. Clin. Lab..

[99] Comabella M., Domínguez C., Rio J., Martín-Gallán P., Vilches A., Vilarrasa N., Espejo C., Montalban X. (2009). Plasma chitotriosidase activity in multiple sclerosis. Clin. Immunol..

[100] Correale J., Fiol M. (2010). Chitinase effects on immune cell response in neuromyelitis optica and multiple sclerosis. Mult. Scler. J..

[101] Lindstedt M. (1991). Multiple sclerosis—Is research on the wrong track?. Med. Hypotheses.

[102] Ramos M., Pisa D., Molina S., Rábano A., Juarranz A., Carrasco L. (2008). Fungal infection in patients with multiple sclerosis. Open Mycol. J..

[103] Napier M.D., Poole C., Satten G.A., Ashley-Koch A., Marrie R.A., Williamson D.M. (2016). Heavy metals, organic solvents and multiple sclerosis: An exploratory look at gene-environment interactions. Arch. Environ. Occup. Health.

[104] Compston A., Lassmann H., McDonald I., Miller D., Noseworthy J., Smith K., Wekerle H., Confavreux C. (2005). The story of multiple sclerosis. Mcalpine’s Multiple Sclerosis.

[105] Parron T., Requena M., Hernandez A.F., Alarcon R. (2011). Association between environmental exposure to pesticides and neurodegenerative diseases. Toxicol. Appl. Pharmacol..

[106] Savage E.P., Keefe T.J., Mounce L.M., Heaton R.K., Lewis J.A., Burcar P.J. (1988). Chronic neurological sequelae of acute organophosphate pesticide poisoning. Arch. Environ. Health.

[107] Bove J., Prou D., Perier C., Przedborski S. (2005). Toxin-induced models of parkinson’s disease. NeuroRx.

[108] Dubey D., Sharma V., Stuve O. (2014). Multiple mothballs or multiple sclerosis: A diagnostic dilemma (p5.192). Neurology.

[109] Oddone E., Crosignani P., Scaburri A., Bai E., Modonesi C., Imbriani M., Bergamaschi R. (2013). Occupation and multiple sclerosis: An italian case-control study. Occup. Environ. Med..

[110] Magyari M., Koch-Henriksen N., Pfleger C.C., Sorensen P.S. (2014). Physical and social environment and the risk of multiple sclerosis. Mult. Scler. Relat. Disord..

[111] Graves J., Chitnis T., Weinstock-Guttman B., Rubin J., Zelikovitch A., Nourbakhsh B., Simmons T., Casper C., Waubant E. (2016). Maternal illness in pregnancy and perinatal exposure to pesticides are associated with risk for pediatric onset ms (s29.005). Neurology.

[112] Landtblom A.-M., Flodin U., Söderfeldt B., Wolfson C., Axelson O. (1996). Organic solvents and multiple sclerosis: A synthesis of the current evidence. Epidemiology.

[113] Iranmanesh F., Ebrahimi H.a., Iranmanesh M., Sedighi B., Gadari F. (2015). Multiple sclerosis and mines: An epidemiologic study from kerman province, Iran. Int. Clin. Neurosci. J..

[114] Razavi Z., Jokar M., Allafchian A., Hossinpour Z., Berenjani L., Shayegan Nejad V. (2016). The relationship between blood lead levels and clinical features among multiple sclerosis patients in Isfahan, Iran. Iran. J. Health, Saf. Environ..

[115] Monti M.C., Guido D., Montomoli C., Sardu C., Sanna A., Pretti S., Lorefice L., Marrosu M.G., Valera P., Cocco E. (2016). Is geo-environmental exposure a risk factor for multiple sclerosis? A population-based cross-sectional study in south-western sardinia. PLoS ONE.

[116] Giacoppo S., Galuppo M., Calabro R.S., D’Aleo G., Marra A., Sessa E., Bua D.G., Potorti A.G., Dugo G., Bramanti P. (2014). Heavy metals and neurodegenerative diseases: An observational study. Biol. Trace Elem. Res..

[117] Fulgenzi A., Zanella S.G., Mariani M.M., Vietti D., Ferrero M.E. (2012). A case of multiple sclerosis improvement following removal of heavy metal intoxication: Lessons learnt from matteo’s case. Biometals.

[118] Crowe W., Allsopp P.J., Watson G.E., Magee P.J., Strain J.J., Armstrong D.J., Ball E., McSorley E.M. (2017). Mercury as an environmental stimulus in the development of autoimmunity—A systematic review. Autoimmun. Rev..

[119] Pamphlett R., Kum Jew S. (2018). Inorganic mercury in human astrocytes, oligodendrocytes, corticomotoneurons and the locus ceruleus: Implications for multiple sclerosis, neurodegenerative disorders and gliomas. Biometals.

[120] Pourahmad J., Kahrizi F., Naderi N., Salimi A., Noorbakhsh F., Faizi M., Naserzadeh P. (2016). Repeated administration of mercury accelerates progression of multiple sclerosis through mitochondrial dysfunction. Iran. J. Pharm. Res..

[121] Guzzi G., Costa A., Pigatto P. (2015). Serum nse and multiple sclerosis. J. Neurol. Sci..

[122] Soni R., Bhatnagar A., Vivek R., Chaturvedi T., Singh A. (2012). A systematic review on mercury toxicity from dental amalgam fillings and its management strategies. J. Sci. Res..

[123] Bjørklund G., Hilt B., Dadar M., Lindh U., Aaseth J. (2018). Neurotoxic effects of mercury exposure in dental personnel. Basic Clin. Pharmacol. Toxicol..

[124] Cariccio V.L., Samà A., Bramanti P., Mazzon E. (2019). Mercury involvement in neuronal damage and in neurodegenerative diseases. Biol. Trace Elem. Res..

[125] Tsai C.-P., Lee C.T.-C. (2013). Multiple sclerosis incidence associated with the soil lead and arsenic concentrations in taiwan. PLoS ONE.

[126] Dehghanifiroozabadi M., Noferesti P., Amirabadizadeh A., Nakhaee S., Aaseth J., Noorbakhsh F., Mehrpour O. (2019). Blood lead levels and multiple sclerosis: A case-control study. Mult. Scler. Relat. Disord..

[127] Alizadeh-Ghodsi M., Zavvari A., Ebrahimi-Kalan A., Shiri-Shahsavar M.R., Yousefi B. (2018). The hypothetical roles of arsenic in multiple sclerosis by induction of inflammation and aggregation of tau protein: A commentary. Nutr. Neurosci..

[128] Jaiser S.R., Winston G.P. (2010). Copper deficiency myelopathy. J. Neurol..

[129] Palm R., Hallmans G. (1982). Zinc and copper in multiple sclerosis. J. Neurol. Neurosurg. Psychiatry.

[130] Bredholt M., Frederiksen J.L. (2016). Zinc in multiple sclerosis: A systematic review and meta-analysis. ASN Neuro.

[131] Zahid Rasul N., Naqab K., Samiullah K., Mehboob A., Mohammad Amjad K. (2018). Potential application of venom proteins in designing of medicines for treating human neurodegenerative disorders. Protein Pept. Lett..

[132] Iwai S., Okazaki M., Kiuchi Y., Oguchi K. (1999). Changes in mrna levels of fibrinogen subunit polypeptides in rats defibrinogenated with batroxobin. Thromb. Res..

[133] Hinman C.L., Stevens-Truss R., Schwarz C., Hudson R.A. (1999). Sequence determinants of modified cobra venom neurotoxin which induce immune resistance to experimental allergic encephalomyelitis: Molecular mechans for immunologic action. Immunopharmacol. Immunotoxicol..

[134] Komegae E.N., Souza T.A.M., Grund L.Z., Lima C., Lopes-Ferreira M. (2017). Multiple functional therapeutic effects of tnp: A small stable synthetic peptide derived from fish venom in a mouse model of multiple sclerosis. PLoS ONE.

[135] Ontaneda D., Hyland M., Cohen J.A. (2012). Multiple sclerosis: New insights in pathogenesis and novel therapeutics. Annu. Rev. Med..

[136] Kamm C.P., Uitdehaag B.M., Polman C.H. (2014). Multiple sclerosis: Current knowledge and future outlook. Eur. Neurol..

[137] Hernan M.A., Alonso A., Hernandez-Diaz S. (2006). Tetanus vaccination and risk of multiple sclerosis: A systematic review. Neurology.

[138] Alonso A., Jick S.S., Jick H., Hernan M.A. (2006). Antibiotic use and risk of multiple sclerosis. Am. J. Epidemiol..

[139] Alonso A., Jick S.S., Hernan M.A. (2006). Allergy, histamine 1 receptor blockers, and the risk of multiple sclerosis. Neurology.

[140] Ifergan I., Wosik K., Cayrol R., Kébir H., Auger C., Bernard M., Bouthillier A., Moumdjian R., Duquette P., Prat A. (2006). Statins reduce human blood-brain barrier permeability and restrict leukocyte migration: Relevance to multiple sclerosis. Ann. Neurol..

[141] Mabunga D.F.N., Gonzales E.L.T., Kim J.-w., Kim K.C., Shin C.Y. (2015). Exploring the validity of valproic acid animal model of autism. Exp. Neurobiol..

[142] Pazhoohan S., Satarian L., Asghari A.A., Salimi M., Kiani S., Mani A.R., Javan M. (2014). Valproic acid attenuates disease symptoms and increases endogenous myelin repair by recruiting neural stem cells and oligodendrocyte progenitors in experimental autoimmune encephalomyelitis. Neurodegener. Dis..

[143] Lv J., Du C., Wei W., Wu Z., Zhao G., Li Z., Xie X. (2012). The antiepileptic drug valproic acid restores t cell homeostasis and ameliorates pathogenesis of experimental autoimmune encephalomyelitis. J. Biol. Chem..

[144] Long J., Chang L., Shen Y., Gao W.H., Wu Y.N., Dou H.B., Huang M.M., Wang Y., Fang W.Y., Shan J.H. (2015). Valproic acid ameliorates graft-versus-host disease by downregulating th1 and th17 cells. J. Immunol..

[145] Askari V.R., Fereydouni N., Baradaran Rahimi V., Askari N., Sahebkar A.H., Rahmanian-Devin P., Samzadeh-Kermani A. (2018). B-amyrin, the cannabinoid receptors agonist, abrogates mice brain microglial cells inflammation induced by lipopolysaccharide/interferon-γ and regulates mφ1/mφ2 balances. Biomed. Pharmacother..

[146] Tanaka M., Moran S., Wen J., Affram K., Chen T., Symes A.J., Zhang Y. (2017). Wwl70 attenuates pge(2) production derived from 2-arachidonoylglycerol in microglia by abhd6-independent mechanism. J. Neuroinflamm..

[147] Guo C., Fulp J.W., Jiang Y., Li X., Chojnacki J.E., Wu J., Wang X.-Y., Zhang S. (2017). Development and characterization of a hydroxyl-sulfonamide analogue, 5-chloro-n-[2-(4-hydroxysulfamoyl-phenyl)-ethyl]-2-methoxy-benzamide, as a novel nlrp3 inflammasome inhibitor for potential treatment of multiple sclerosis. ACS Chem. Neurosci..

[148] Leonoudakis D., Rane A., Angeli S., Lithgow G.J., Andersen J.K., Chinta S.J. (2017). Anti-inflammatory and neuroprotective role of natural product securinine in activated glial cells: Implications for parkinson’s disease. Med. Inflamm..

[149] Kappos L., Gold R., Miller D.H., MacManus D.G., Havrdova E., Limmroth V., Polman C.H., Schmierer K., Yousry T.A., Yang M. (2008). Efficacy and safety of oral fumarate in patients with relapsing-remitting multiple sclerosis: A multicentre, randomised, double-blind, placebo-controlled phase iib study. Lancet.

[150] Stangel M., Linker R.A. (2013). Dimethyl fumarate (bg-12) for the treatment of multiple sclerosis. Expert Rev. Clin. Pharmacol..

[151] Linker R.A., Gold R. (2013). Dimethyl fumarate for treatment of multiple sclerosis: Mechanism of action, effectiveness, and side effects. Curr. Neurol. Neurosci. Rep..

[152] Bomprezzi R. (2015). Dimethyl fumarate in the treatment of relapsing–remitting multiple sclerosis: An overview. Ther. Adv. Neurol. Disord..

[153] Gopal S., Mikulskis A., Gold R., Fox R.J., Dawson K.T., Amaravadi L. (2017). Evidence of activation of the nrf2 pathway in multiple sclerosis patients treated with delayed-release dimethyl fumarate in the phase 3 define and confirm studies. Mult. Scler..

[154] Mills E.A., Ogrodnik M.A., Plave A., Mao-Draayer Y. (2018). Emerging understanding of the mechanism of action for dimethyl fumarate in the treatment of multiple sclerosis. Front. Neurol..

[155] Vego H., Sand K.L., Høglund R.A., Fallang L.-E., Gundersen G., Holmøy T., Maghazachi A.A. (2014). Monomethyl fumarate augments nk cell lysis of tumor cells through degranulation and the upregulation of nkp46 and cd107a. Cell Mol. Immunol..

[156] Maghazachi A.A., Sand K.L., Al-Jaderi Z. (2016). Glatiramer acetate, dimethyl fumarate, and monomethyl fumarate upregulate the expression of ccr10 on the surface of natural killer cells and enhance their chemotaxis and cytotoxicity. Front. Immunol..

[157] Al-Jaderi Z., Maghazachi A.A. (2016). Utilization of dimethyl fumarate and related molecules for treatment of multiple sclerosis, cancer, and other diseases. Front. Immunol..

[158] Strader C.R., Pearce C.J., Oberlies N.H. (2011). Fingolimod (fty720): A recently approved multiple sclerosis drug based on a fungal secondary metabolite. J. Nat. Prod..

[159] Al-Jaderi Z., Maghazachi A.A. (2013). Effects of vitamin d3, calcipotriol and fty720 on the expression of surface molecules and cytolytic activities of human natural killer cells and dendritic cells. Toxins (Basel).

